# Human Infection with Marten Tapeworm

**DOI:** 10.3201/eid1907.121114

**Published:** 2013-07

**Authors:** Philipp Eberwein, Alexandra Haeupler, Fabian Kuepper, Dirk Wagner, Winfried V. Kern, Birgit Muntau, Paul Racz, Hansjuergen Agostini, Sven Poppert

**Affiliations:** Albert-Ludwigs-University, Freiburg, Germany (P. Eberwein, F. Kuepper, D. Wagner, W.V. Kern, H. Agostini);; Bernhard Nocht Institute for Tropical Medicine, Hamburg, Germany (A. Haeupler, B. Muntau, P. Racz, S. Poppert)

**Keywords:** taenia, cysticercosis, *Taenia martis*, marten, helminth, cestode, tapeworm, human, parasites

**To the Editor:** Cysticercosis-like human infections with the tapeworm *Taenia crassiceps*, which infects foxes as terminal hosts, have been reported (*1*,*2*). We report a case of a cysticercosis-like eye infection caused by the tapeworm *T. martis* (marten tapeworm) in a woman.

The patient was a 43-year-old German woman who sought care during July 2010, after 4 days of perceiving flashing lights in her visual field and a paracentral scotoma in her left eye. Visual acuity in both eyes was 20/20. Examination of the left fundus revealed a mobile subretinal tumor at the temporal upper retinal branch vessel with adjacent intraretinal and subhyaloid bleeding (Figure, panels A–C; Video). The subretinal tumor resembled a cestode larva. 

**Figure:**
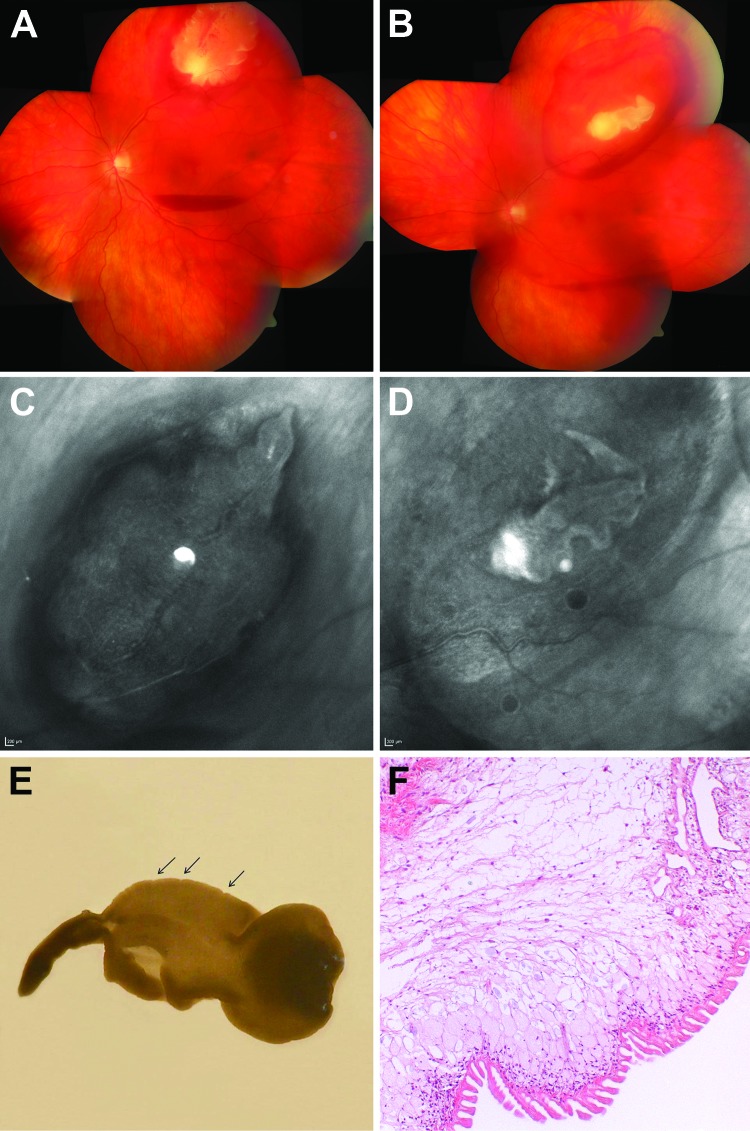
Cysticercosis-like eye infection caused by the tapeworm *Taenia martis* in a woman. A) Fundus at the patient’s initial visit, before medical therapy. The cyst lies subretinally at the temporal upper branch vessels; adjacent intraretinal and subretinal bleeding and central subhyaloid bleeding can be seen. B) After 8 days of medical therapy, the cyst size had decreased markedly. The physis of the larva (A and B) is reminiscent of the armatetrathyridium (or fimbriocercus), a larval form typical for the tapeworm subspecies *T. martis martis.* C) Cyst at patient’s initial visit. D) Cyst at the time of surgery. E) Surgically removed monocephalic cysticercus-like larva with inverted parenchymatous portion, withdrawn scolex, and attenuated posterior end. The tegumental surface is transversely striated and exhibits inward folds (arrows). F) Histologic section of the *Taenia martis* tapeworm cyst showing morphologic characteristics also commonly seen in cysticercosis cysts caused by *T. solium* tapeworms. The syncytial bladder wall consists of a rugate external, a nucleated intermediate, and an internal reticular layer with lacunate branches of the excretory duct system. Filamentous extensions of contractile muscles project into the parenchyma, which is interspersed with a few calcareous corpuscles. In addition, the *T. martis* cyst shows a preponderance of uniformly organized, elongate and slender tegumental processes, which are usually not seen in histologic sections of cyst walls caused by *T. solium* tapeworms. Hematoxylin and eosin stain; objective magnification ×10.

**Video:**
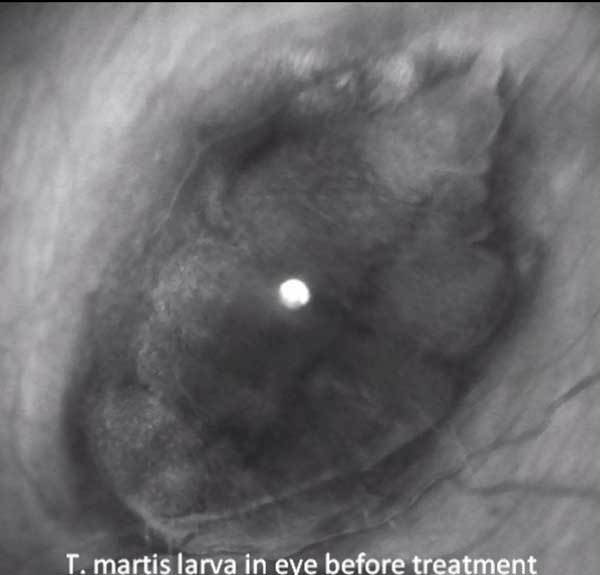
*Taenia martis* tapeworm cyst in woman with cysticercosis–like eye infection, before and after medical therapy. Although the cyst decreased in size after medical therapy, movements of the larva persisted. Video is 100 seconds long, taken with a Heidelberg Retinal Analyzer.

Direct Video Link: http://streaming.cdc.gov/vod.php?id=eff01b6f9bbe41f1ea318db3d6e66f9220121218092846890

The patient reported no other symptoms at that time. Laboratory evaluation found no eosinophilia or elevation of total IgE. Serologic testing results were negative for antibodies against the following parasites: *Taenia solium*, *Echinococcus multilocularis,* *E. granulosus,* *Dirofilaria immitis, Strongyloides* spp., and *Toxocara canis.* Fecal testing results were negative for worm eggs. Images from ultrasonography of the liver and magnetic resonance imaging of the head were unremarkable. The patient’s travel history included—in addition to southern European countries—trips to Nepal and Thailand 15 years previously. 

At the time of examination, the patient lived in a small village near Freiburg (im Breisgau) in southwestern Germany. She grew vegetables in the family garden, which was next to a forest. Her 3 children and husband did not report any health problems. For the past 6 years, the family had owned a dog, which received antiparasitic medications on a regular basis; recent checks for intestinal parasitic infection found no ova.

The suspected cause of the woman’s illness was cysticercosis caused by the larva of *T. solium*; systemic antiparasitic therapy was started (albendazole 400 mg 2×/d, dexamethasone 20 mg/d). The size of the larva diminished (Figure, panel D; Video), but the patient remained symptomatic. Therefore, after 8 days of therapy, the cyst was removed by retinotomy. A few days later, peripheral retinal detachment occurred and was treated by a second vitrectomy and intravitreal gas injection. Because of the repeated gas tamponade, a gas cataract developed, which necessitated cataract surgery. At the end of March 2011, the patient’s visual acuity had returned to 20/20 in both eyes.

The removed cyst showed the characteristic macroscopic and histologic features of a cysticercus bladder wall (Figure, panels E, F). To determine the exact species by using molecular methods, we isolated DNA from the cyst, conducted different PCRs selective for mitochondrial genes, determined the corresponding sequences, and used a BLAST search (*3*) to compare these sequences with publically available sequences. Sequences of the following mitochondrial genes were determined by using the given primers and later submitted to GenBank: small ribosomal subunit (primers 12S *Taenia* FF 5′-CACAGTGCCAGCATCYGCGGT-3′ and 12S *Taenia* RR 5′-GAGGGTGACGGGCGGTGTGTAC-3′, PCR product of 426 bp, GenBank accession no. JX415820); NADH dehydrogenase subunit 1 (primers: NAD1-FF 5′-ATTGGKTTATTTCAGAGTTTTTCTGATTTA-3′ and NAD1-RR 5′-CTCMCCATAATCAAATGGACTACG-3′, 394 bp, GenBank accession no. JX415819); and the cytochrome-*c* oxidase subunit 1 (determined by using previously published primers [*4**,**5*]; 376 bp, GenBank accession no. JX415821). All sequences showed highest identity with *T. martis* (99%–100%) but substantially lower identity with *T. crassiceps* (91%–97%) and *T. solium* (87%–89%) tapeworms.

Thus, molecular methods unequivocally identified the larva as that of a *T. martis* tapeworm. *T. martis* tapeworms (cestodes) live and produce eggs in the intestines of definitive hosts, weasels (family *Mustelidae*), which also includes pine martens, stone martens, polecats, badgers, wolverines, and stoats (*6*). The intermediate hosts are prey animals of the definitive hosts, such as arvicoline (voles and muskrats) or murid rodents. When intermediate hosts ingest eggs, cysticerci develop in the pleural and peritoneal cavities. *T. martis* tapeworms probably occur worldwide wherever suitable definitive and intermediate hosts are present (*6*,*7*). A study in southwest Germany reported that 36% of stone martens were infected with *T. martis* tapeworms (*6*).

Although nearly all patients who had cysticercosis-like infections caused by *T. crassiceps* tapeworms were immunosuppressed (*1*,*2*), we found no signs of immunosuppression in the patient reported here. The only identified risk factor for this patient was consumption of homegrown vegetables, which could have been contaminated by marten feces.

The clinical and histologic appearance of the organism in this patient suggested cysticercosis caused by a *T. solium* tapeworm. However, the specific diagnosis of *T. martis* tapeworm infection was possible only by use of molecular methods. Thus, human infections with *T. martis* and other animal tapeworms might occur at times but might be misdiagnosed as *T. solium* cysticercosis. For therapy, the rules and considerations are probably the same as those for *T. solium* cysticercosis, as described (*8*,*9*). Concerning antiparasitic therapy, one must be aware of possible complications caused by intraocular immunologic reactions. As demonstrated by the case reported here, surgical removal of a subretinal larva is connected with the risk for retinal detachment and cataract formation. The identification of the responsible tapeworm is useful for epidemiologic reasons, for determining the source of infection. We therefore suggest using molecular methods to determine the exact species of parasites removed from human tissue.
